# ApoE4 attenuates autophagy via FoxO3a repression in the brain

**DOI:** 10.1038/s41598-021-97117-6

**Published:** 2021-09-02

**Authors:** Hee-Young Sohn, Seong-Ik Kim, Jee-Yun Park, Sung-Hye Park, Young Ho Koh, Joon Kim, Chulman Jo

**Affiliations:** 1 Division of Brain Disease Research, Department for Chronic Disease Convergence Research, Korea National Institute of Health, 187 Osongsaengmyeong2-ro, Osong-eup, Cheongju-si, Chungcheongbuk-do 28159 Republic of Korea; 2 Department of Pathology, Seoul National University College of Medicine, Seoul, 03080 Republic of Korea; 3 Laboratory of Biochemistry, Division of Life Sciences, Korea University, Seoul, 02841 Republic of Korea

**Keywords:** Molecular biology, Neuroscience, Neurology

## Abstract

Apolipoprotein E (ApoE) plays multiple roles in lipid transport, neuronal signaling, glucose metabolism, mitochondrial function, and inflammation in the brain. It is also associated with neurodegenerative diseases, and its influence differs depending on the isoform. In particular, the ε4 allele of *APOE* is the highest genetic risk factor for developing late-onset Alzheimer’s disease (AD). However, the mechanism by which ApoE4 contributes to the pathogenesis of AD remains unclear. We investigated the effect of ApoE4 on autophagy in the human brains of ApoE4 carriers. Compared to non-carriers, the expression of FoxO3a regulating autophagy-related genes was significantly reduced in ApoE4 carriers, and the phosphorylation level of FoxO3a at Ser253 increased in ApoE4 carriers, indicating that FoxO3a is considerably repressed in ApoE4 carriers. As a result, the protein expression of FoxO3a downstream genes, such as Atg12, Beclin-1, BNIP3, and PINK1, was significantly decreased, likely leading to dysfunction of both autophagy and mitophagy in ApoE4 carriers. In addition, phosphorylated tau accumulated more in ApoE4 carriers than in non-carriers. Taken together, our results suggest that ApoE4 might attenuate autophagy via the repression of FoxO3a in AD pathogenesis. The regulation of the ApoE4-FoxO3a axis may provide a novel therapeutic target for the prevention and treatment of AD with the *APOE4* allele.

## Introduction

Apolipoprotein E (ApoE) is a multifunctional glycoprotein that plays a major role in redistributing cholesterol and other lipids by binding to cell-surface ApoE receptors such as LDL receptor (LDLR) and LDL receptor-related protein 1 (LRP1) in the brain^1^. In addition to lipid transport, ApoE modulates multiple pathways in the brain, including synaptic plasticity, glucose metabolism, mitochondrial function, and cerebrovascular function^2–4^. There are three ApoE isoforms (ApoE2, ApoE3, and ApoE4) in humans that have different effects on biological processes^5,6^. Among the three isoforms, the *APOE4* allele is associated with an increased risk of Alzheimer’s disease (AD) and a lower age of onset^7^. AD is pathologically characterized by the presence of extracellular amyloid plaques and intracellular neurofibrillary tangles composed of phosphorylated tau protein, as well as extensive neuronal and synaptic losses^8,9^. Growing evidence suggests that the effect of ApoE4 on AD risk is exerted through either the clearance inhibition or aggregation promotion of pathological tau as well as amyloid-beta^3,10,11^; however, the exact etiology of ApoE4 in AD remains elusive.

Macroautophagy (hereafter referred to as autophagy) is a conserved intracellular process that clears aggregated proteins and damaged cellular organelles^12^. Aberrant autophagy contributes to protein aggregation, organelle impairment, and neuronal loss, ultimately leading to neurodegenerative diseases such as AD, Parkinson’s disease, and Huntington’s disease^12,13^. In particular, the number of autophagic vacuoles, an intermediate double-membraned vesicle in the autophagy pathway, is higher in the brains of individuals with neurodegenerative diseases than in those of healthy controls suggesting an impairment in the maturation of autophagosomes to autolysosomes^4,14^. Mitochondrial dysfunction and impairment of mitophagy have also been implicated in aging and neurodegenerative diseases such as AD^15,16^.

Mitophagy, selective autophagy of mitochondria, is an important cellular process for quality control and clearance of mitochondria^16^. Mitophagy is mediated by either the PTEN-induced serine/threonine kinase (PINK1)-Parkin pathway or the receptor-mediated pathway^16^. PINK1 senses mitochondrial damage and phosphorylates ubiquitin at Ser65^17^. P-ubiquitin (Ser65) triggers parkin activation, and then autophagy receptor proteins, including optineurin, NDP52, and p62, are recruited. These proteins mediate the degradation of damaged mitochondria by interacting with the autophagosomal protein LC3 or gamma-aminobutyric acid receptor-associated protein (GABARAP)^16,18,19^. Some outer mitochondrial membrane proteins, such as BCL2-interacting protein 3 (BNIP3) and Nip3-like protein X (NIX), promote mitophagy under stress conditions in an independent manner of the PINK1-Parkin pathway^16,20^.

FoxO transcription factors mainly function to maintain cellular and organismal homeostasis, especially in response to stresses such as oxidative and metabolic stress ^21,22^. In mammals, there are four FoxOs: FoxO1, FoxO3a, FoxO4, and FoxO6^23^. Among them, FoxO3a is highly expressed in the brain and controls multiple metabolic pathways for neuronal protection^21^. In addition, FoxO3a is associated with the transcriptional regulation of autophagy in various tissues as well as in adult neural stem cells^24–26^. It activates the transcription of several autophagy genes, such as *ATG12*, *VPS34*, and *BECN1*, as well as mitophagy genes *BNIP3* and *PINK1*^24,27^. FoxO activity is controlled by their subcellular localization, and multiple Ser/Thr kinases such as AKT, serum and glucocorticoid-induced kinase, and AMPK signaling pathways are involved in the translocation of FoxOs^23^. In the regulation of FoxO3a, AKT can phosphorylate FoxO3a at Thr32, Ser253, and Ser315 residues, leading to the inhibition of FoxO3a activity via the docking of 14-3-3 and exclusion of FoxO3a from the nucleus^24,28^.

Several lines of accumulating evidence suggest that FoxO3a positively regulates the expression of several autophagy- and mitophagy-related genes, thus protecting cells against protein toxicity from the aggregation of misfolded or abnormal proteins^25,26,29,30^. In this study, we examined the protein levels of FoxO3a and its downstream genes in the human brain tissues of elderly ApoE4 carriers. We found that the expression levels of FoxO3a and its autophagy- and mitophagy-related downstream genes were significantly lower in ApoE4 carriers than in non-carriers. Thus, our results suggest that ApoE4 plays a crucial role in AD pathology, such as dysfunction of autophagy and mitophagy through the repression of FoxO3a.

## Results

### FoxO3a was repressed in the brains of ApoE4 carriers

**Figure 1:**
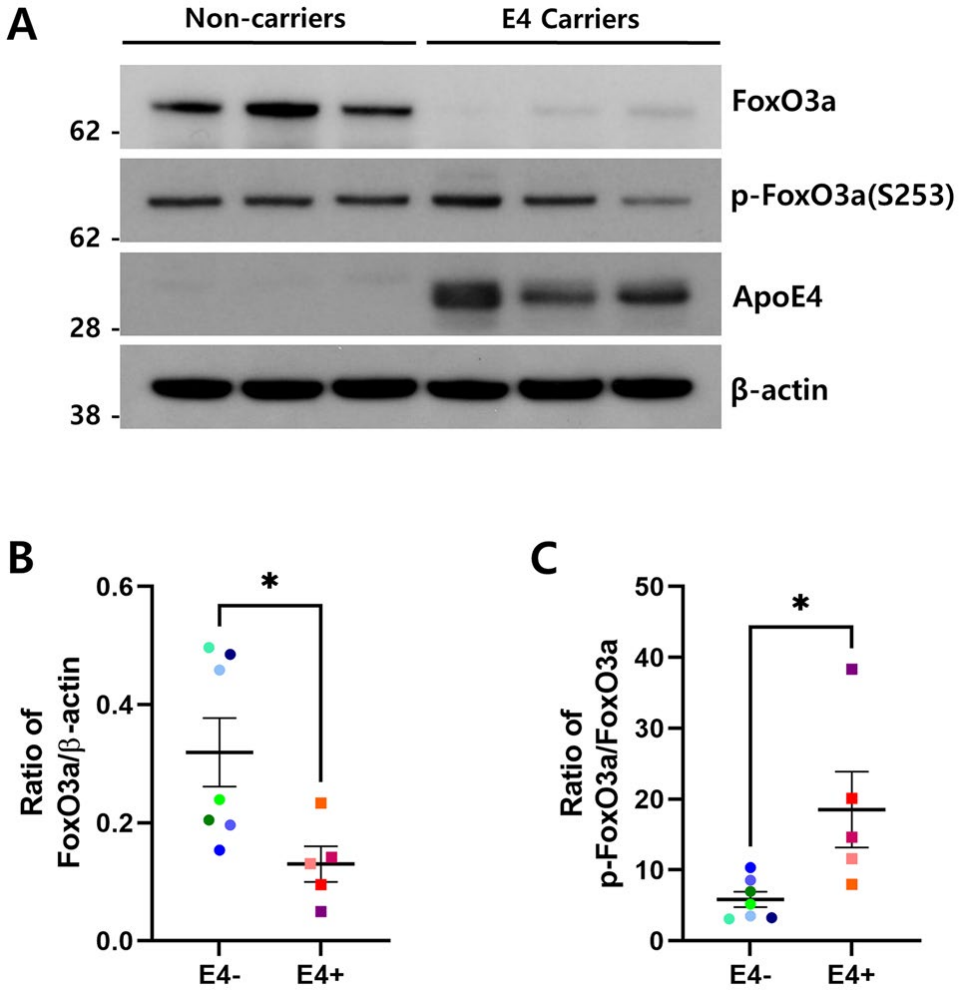
FoxO3a was repressed in human brains of ApoE4 carriers. Human postmortem brain tissues (superior temporal gyrus) were collected to compare ApoE4 non-carriers (n = 7, E4−) vs. carriers (n = 5, E4 +). The protein levels of FoxO3a, p-FoxO3a (Ser253), ApoE4, and β-actin were analyzed by immunoblotting using the corresponding antibodies, respectively. (**A**) shows the representative western blot analysis of FoxO3a and p-FoxO3a (Ser253). β-actin was used as a loading control. Full blots are provided in Supplementary Fig. S4. Relative ratio of FoxO3a (**B**) and p-FoxO3a (Ser253) (**C**) to the protein level of β-actin and FoxO3a, respectively. Data shown are mean ± SEM and were analyzed using the Student’s t-test. (*p < 0.05).

Previous studies have reported that ApoE4 is associated with dysfunction of autophagy in the brain, and the transcription factor FoxO3a acts as a regulator of autophagy in multiple cells^24,31,32^. Based on this, we hypothesized that ApoE4 influences autophagy via FoxO3a. Therefore, we investigated the expression level of FoxO3a protein in human brain tissues of ApoE4 carriers and compared them with those in non-carriers. As shown in Fig. 1A,B, the expression level of FoxO3a protein was significantly lower in ApoE4 carriers than in non-carriers (*p* < 0.05). Intracellular localization is important for FoxO3a transcriptional activity. The phosphorylation of FoxO3a inhibits its transcriptional activity by sequestering it in the cytoplasm^23^. Therefore, we examined the level of phosphorylated FoxO3a at Ser253 between ApoE4 carriers and non-carriers. The level of phosphorylated FoxO3a at Ser253 was dramatically higher in ApoE4 carriers than in non-carriers (*p* < 0.05) (Fig. 1A,C). Together, these results indicate that ApoE4 inhibits the expression and transcriptional activity of FoxO3a.

### Autophagy-related gene products decreased in ApoE4 carriers

**Figure 2:**
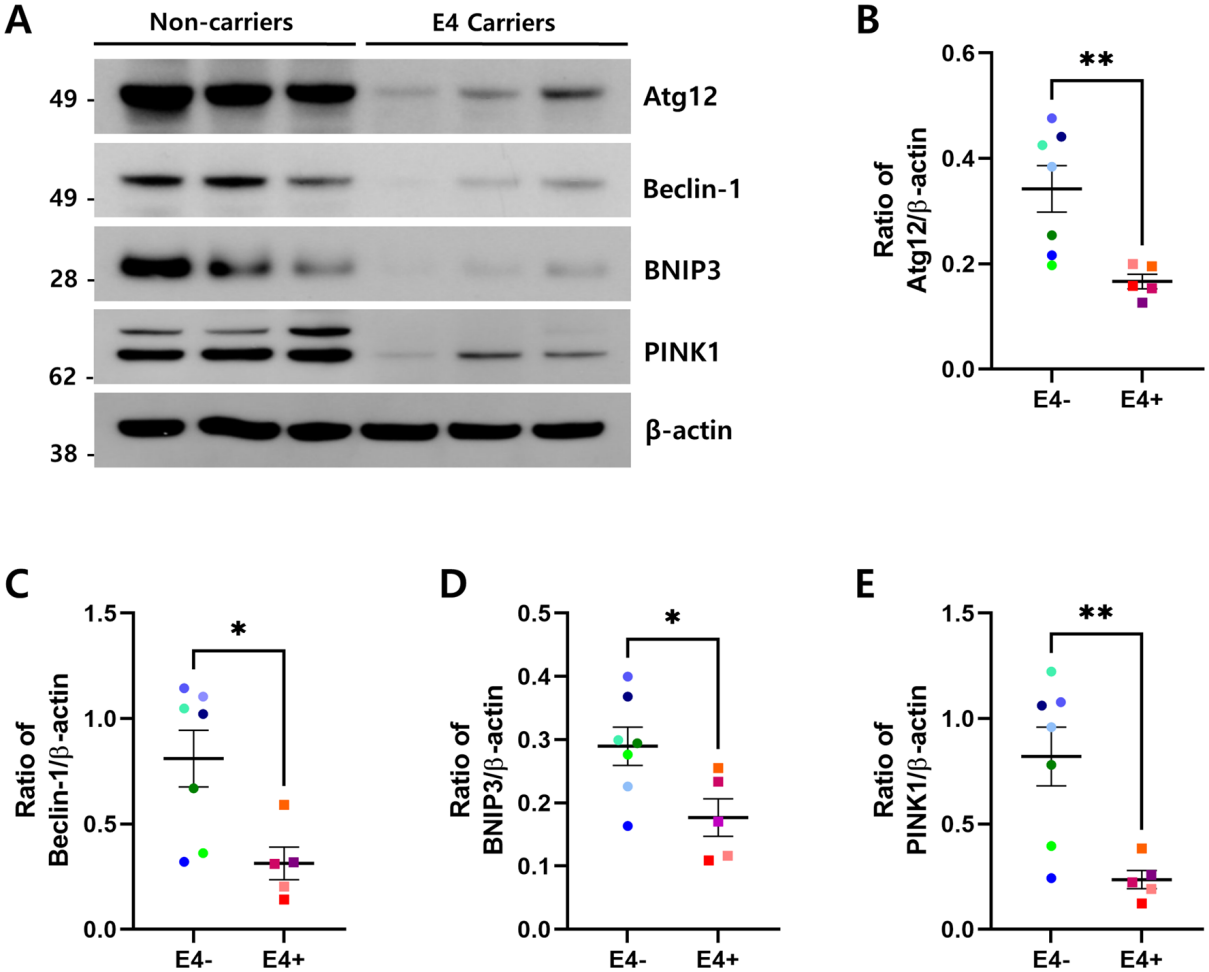
Protein levels of FoxO3a downstream genes decreased in ApoE4 carriers. The protein levels of Atg12, Beclin-1, BNIP3, PINK1, and β-actin in ApoE4 non-carriers (n = 7, E4−) vs. carriers (n = 5, E4 +) were analyzed by immunoblotting using the corresponding antibodies, respectively. (**A**) shows the representative western blot analysis of Atg12, Beclin-1, BNIP3, and PINK1. β-actin was used as a loading control. Full blots are provided in Supplementary Fig. S4. Relative ratio of Atg12 (**B**), Beclin-1 (**C**), BNIP3 (**D**) and PINK1 (**E**) against the protein level of β-actin. Data shown are mean ± SEM and were analyzed using the Student’s t-test. (*p < 0.05, **p < 0.01).

We further tested whether the repression of FoxO3a affects the expression of downstream genes related to autophagy and mitophagy. The expression levels of Atg12, Beclin-1, BNIP3, and PINK1 proteins were analyzed by immunoblotting (Fig. 2A). Compared with non-carriers, the expression levels of Atg12, Beclin-1, BNIP3, and PINK1 proteins were significantly reduced in ApoE4 carriers (Fig. 2B–E). To confirm whether the decrease in these proteins was caused by the suppression of FoxO3a activity, we conducted correlation analyses for the levels of FoxO3a and Atg12, Beclin-1, BNIP3, and PINK1. Positive correlations were observed in all subjects (Supplementary Fig. S2). Together, our data suggest that ApoE4 significantly decreases autophagy/mitophagy component proteins via the repression of FoxO3a, which likely leads to the alteration of both autophagy and mitophagy.

### Autophagy/mitophagy were dysregulated in ApoE4 carriers

**Figure 3:**
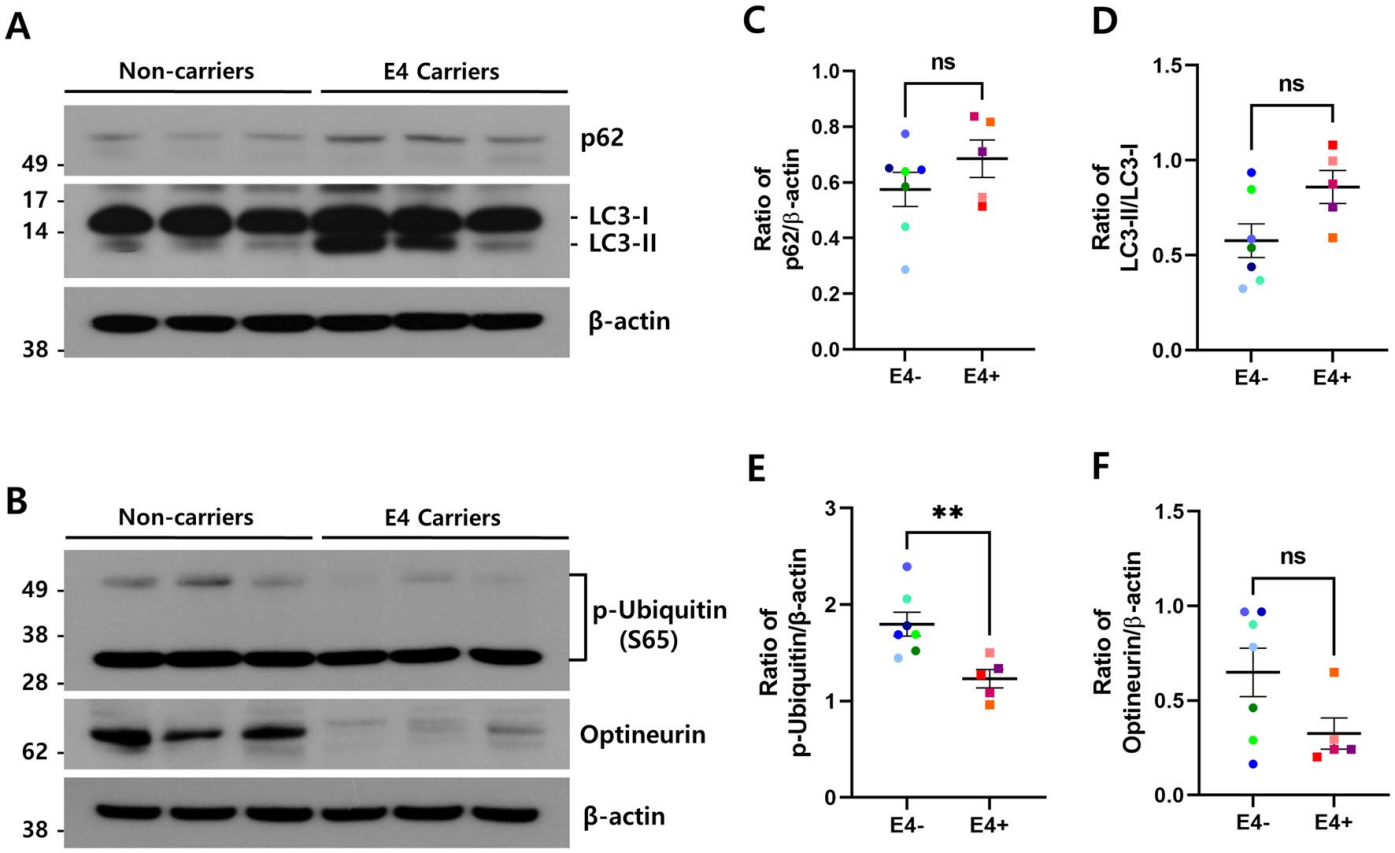
Autophagy/mitophagy components were quantitatively altered in the brains of ApoE4 carriers. The protein levels of p62, LC3, p-ubiquitin (Ser65), optineurin, and β-actin in ApoE4 non-carriers (n = 7, E4-) vs. carriers (n = 5, E4 +) were analyzed by immunoblotting using corresponding antibodies, respectively. Representative western blot analysis of p62 and LC3 (**A**), p-ubiquitin (Ser65), and optineurin (**B**). β-actin was used as a loading control. Full blots are provided in Supplementary Fig. S4. Relative ratio of p62 (**C**), LC3-II (**D**) p-ubiquitin (Ser65) (**E**), and optineurin (**F**) against the protein levels indicated on the y-axis, respectively. Data shown are mean ± SEM. (**p < 0.01). *ns* not significant.

To further examine the autophagic status, we analyzed the expression levels of autophagy markers LC3 and p62 (Fig. 3A). The ratio of LC3-II/LC3-I was increased in the brains of ApoE4 carriers compared with non-carriers (Fig. 3D). Although the conversion ratio of LC3-I to LC3-II is used as a measure of autophagy activation, an increase in autophagosomes has been observed in the brains of AD patients through autophagy dysfunction^33^. This suggests that the impairment of maturation in autophagosome to lysosome fusion could induce the accumulation of autophagosomes with LC3-II, thus increasing the autophagosomal marker LC3-II. In addition, p62 protein levels also increased (Fig. 3C). Because p62 itself is degraded during autophagy, it is speculated that the increase of p62 protein along with LC3-II might be resulted from autophagy dysregulation in ApoE4 carriers.

To assess the status of mitophagy in the brains of ApoE4 carriers, we examined the expression levels of p-ubiquitin, which is a substrate of PINK1, and optineurin, a well-known mitophagy receptor protein, by immunoblotting (Fig. 3B). As shown in Fig. 3E, the phosphorylation level of the mitophagy activation marker ubiquitin was significantly reduced in ApoE4 carriers compared with non-carriers (*p* < 0.01), indicating that the activity of PINK1 decreased in ApoE4 carriers. In addition, the level of the autophagy receptor optineurin also decreased in ApoE4 carriers (Fig. 3F). The reduction of p-ubiquitin and optineurin indicated that the mitophagy pathway was also suppressed in ApoE4 carriers. Together, these data suggest that ApoE4 might influence the dysfunction of both autophagy and mitophagy via the repression of FoxO3a.

### Accumulation of phosphorylated tau increased in ApoE4 carriers

**Figure 4:**
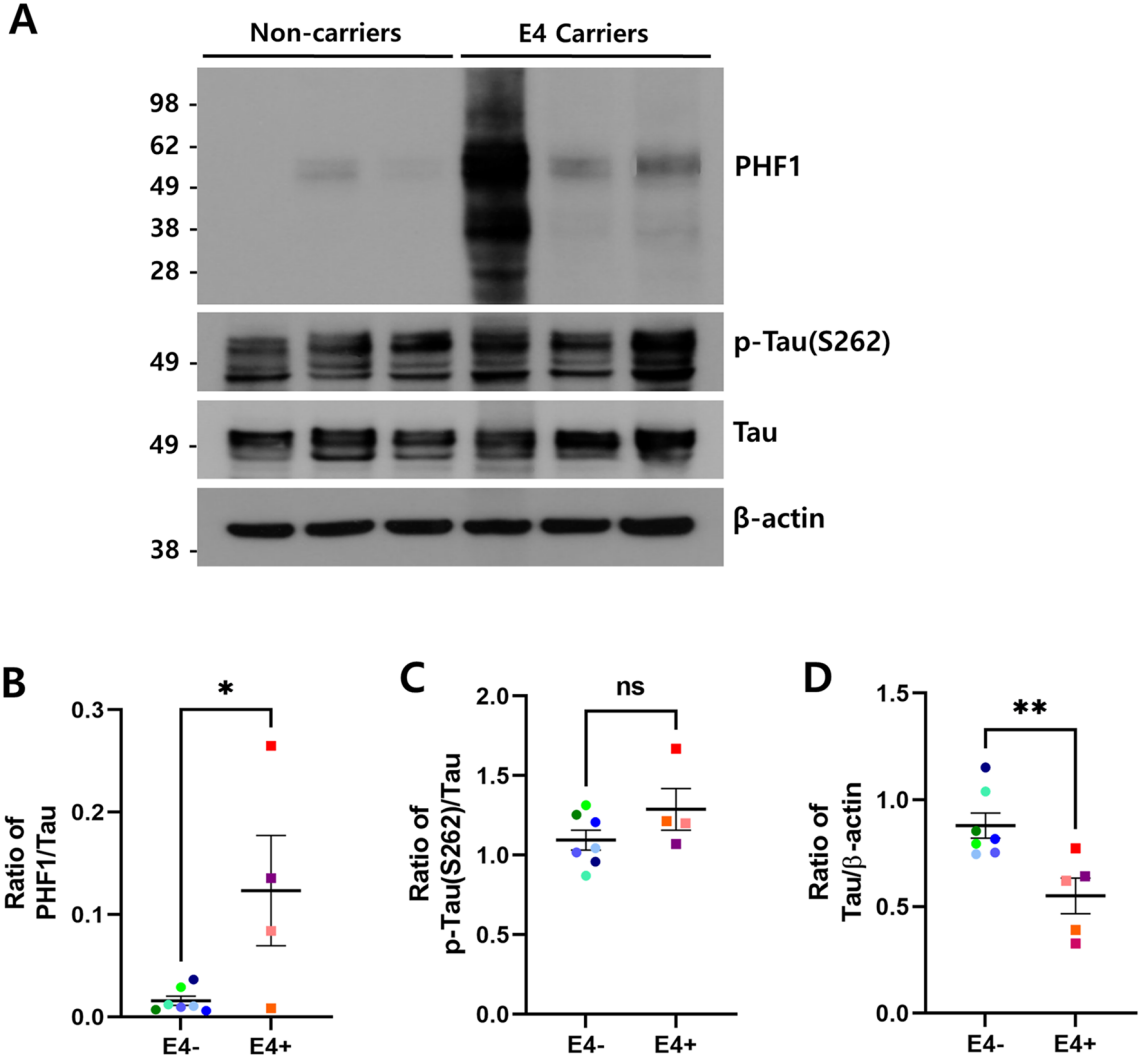
Phosphorylated tau increased in the brain of ApoE4 carriers. The protein levels of phosphorylated tau, total tau, and β-actin in ApoE4 non-carriers (n = 7, E4−) vs. carriers (n = 5, E4 +) were analyzed by immunoblotting using the corresponding antibodies, respectively. (**A**) shows the representative western blot analysis of tau phosphorylated at Ser396/404 (PHF1) and Ser262 (p-tau [S262]) and total tau proteins. β-actin was used as a loading control. Full blots are provided in Supplementary Fig. S4. Relative ratio of tau phosphorylated at Ser396/404 (**B**) and Ser262 (**C**), and total tau (**D**) to the protein levels of tau and β-actin, respectively. All data are presented as mean ± SEM. (*p < 0.05, **p < 0.01). *ns* not significant.

**Figure 5:**
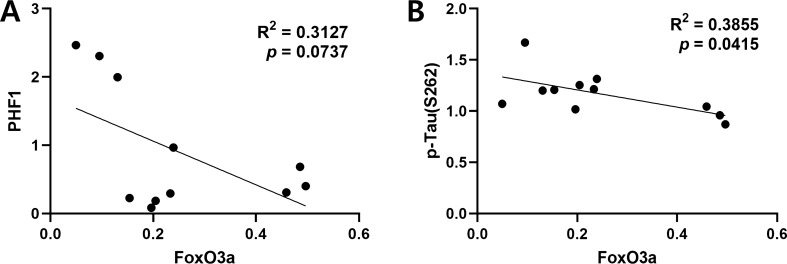
Protein levels of phosphorylated tau were negatively correlated with those of FoxO3a. (**A**) The correlation of protein levels between FoxO3a and tau phosphorylated at S396/404 (PHF1) was analyzed with the Pearson’s correlation test (E4 carriers n = 4, non-carriers n = 7). (**B**) The correlation of protein levels between FoxO3a and tau phosphorylated at S262 protein levels was analyzed with the Pearson’s correlation test (E4 carriers n = 4, non-carriers n = 7). Linear regressions are shown as solid lines.

To examine the effect of FoxO3a reduction on tau pathology in ApoE4 carriers, we measured the levels of phosphorylated tau and total tau proteins by immunoblotting (Fig. 4A). Among the 12 cases, one case of ApoE4 carriers was a vascular dementia (VD) patient. Because the pathology of patients with VD is largely different from that of AD, it was excluded from the quantification and correlation analysis of tau pathology. As shown in Fig. 4B, the levels of tau phosphorylated at Ser396/404 epitopes detected by a PHF1 antibody were significantly increased in ApoE4 carriers compared with non-carriers (*p* < 0.05). Moreover, the level of tau phosphorylated at Ser262 also increased (Fig. 4C). In contrast, the expression level of total tau protein was lower in ApoE4 carriers compare to non-carriers (*p* < 0.01; Fig. 4D). Interestingly, the Braak stage, which indicates the degree of tau pathology, was higher in ApoE4 carriers than in non-carriers compared to AD patients of similar age (Supplementary Table). Together, these results suggest that ApoE4 may accelerate the progression of tau pathophysiology. Furthermore, we performed a correlation analysis between the levels of FoxO3a and phosphorylated tau proteins. As shown in Fig. 5A, the level of phosphorylated tau at Ser396/404 residues was negatively correlated with that of FoxO3a (R^2^ = 0.3127; *p* = 0.0737; n = 11). The level of phosphorylated tau at Ser262 also showed a negative, significant correlation with that of FoxO3a (R^2^ = 0.3855; *p* = 0.0415; n = 11; Fig. 5B). These results demonstrate that the repression of FoxO3a might contribute to the accumulation of phosphorylated tau proteins in ApoE4 carriers.

## Discussion

**Figure 6:**
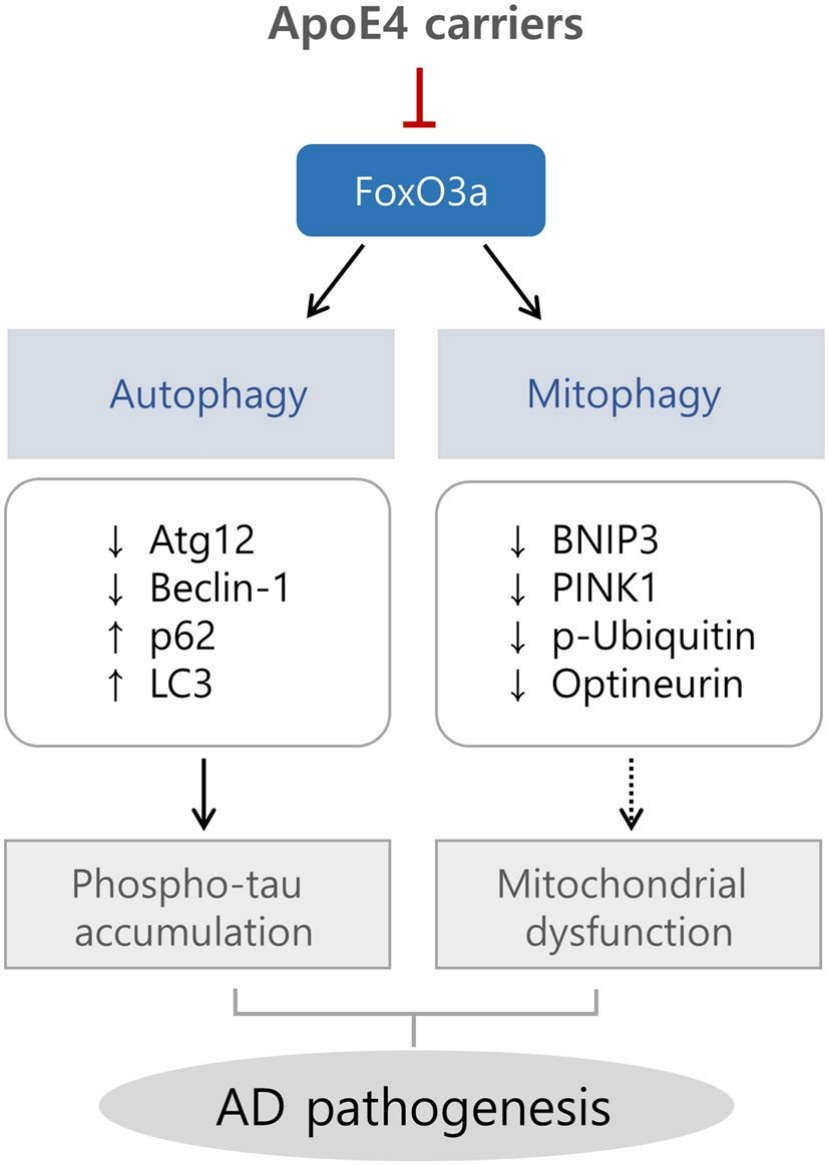
Schematic diagram of the relationship between ApoE4 and FoxO3a in AD pathogenesis. ApoE4 represses the transcription factor FoxO3a. Downregulation of FoxO3a reduces the protein levels of its autophagy/mitophagy-related downstream genes, such as Atg12, Beclin-1, BNIP3, and PINK1, which could result in an impairment of autophagy and mitophagy. Dysfunction of autophagy might increase the levels of phosphorylated tau proteins, leading to AD pathogenesis.

Proteostasis is a process of protein homeostasis that maintains the quality control of proteins and organelles from synthesis to degradation, and autophagy is an intracellular degradation pathway that removes misfolded and proteotoxic proteins^34,35^. Impaired autophagy in the brain is a common feature of aging and neurodegenerative diseases^36,37^. Therefore, modulation of autophagy is important for preventing the occurrence and progression of neurodegenerative diseases. In this study, we showed that ApoE4 represses FoxO3a, resulting in decreased protein expression of autophagy- and mitophagy-related genes that are regulated by FoxO3a. We suggest that the reduction of these autophagy-essential proteins might induce dysfunction of autophagy and mitophagy and subsequently lead to an increase in phosphorylated tau proteins in ApoE4 carriers (Fig. 6). A previous study observed that serum FoxO3a was significantly lower in AD than in mild cognitive impairment and geriatric control^38^. Moreover, it was found that mitochondrial biogenesis, dynamics, and antioxidant response proteins were downregulated in ApoE4 carriers relative to non-carriers, and the level of FoxO3a was lowered in postmortem human brains^39^. These findings are consistent with our work showing that the repression of FoxO3a by ApoE4 might be involved in AD pathology.

ApoE4 is involved in autophagy dysfunction^31,32^. Simonovitch et al. demonstrated that autophagy is impaired in ApoE4 astrocytes^31^. Furthermore, a recent study suggested that ApoE4 inhibits the expression of autophagy-related genes by competitively binding ApoE4 to the coordinated lysosomal expression and regulation (CLEAR) DNA elements in which transcription factor EB (TFEB), a master regulator of autophagy and lysosome biogenesis, normally induces its downstream genes^32^. Here, we added FoxO3a as an intermediator in ApoE4-mediated autophagy dysfunction. As shown in Fig. 1, ApoE4 strongly represses the expression of FoxO3a protein. Therefore, it is speculated that ApoE4 inhibits FoxO3a expression especially in the transcription level. Therefore, we analyzed whether the CLEAR element is in FoxO3a promoter region. We found a CLEAR element in the promoter region of human FoxO3a (data not shown). Thus, it is possible that ApoE4 competitively inhibits the binding of TFEB to the CLEAR element in the promoter of FoxO3a, thus repressing the expression of FoxO3a. In addition, we examined the association between ApoE and FoxO3a using the co-immunoprecipitation technique. Interestingly, the amount of FoxO3a co-immunoprecipitated with ApoE4 was increased than that with ApoE3 (Supplementary Fig. S3). Thus, how this interaction could affect the repression of FoxO3a remains to be investigated.

ApoE4 is known to be associated with tau pathology in AD^10,40,41^. Shi et al. demonstrated that ApoE4 promotes tau pathology, neuroinflammation, and tau-mediated neurodegeneration relative to other ApoE isoforms^10^. Kang et al. reported that ApoE4 exacerbates tau pathology via inhibition of vesicular monoamine transporter_2_ (VMAT_2_) in the locus coeruleus, reduces hippocampal volume, and induces cognitive dysfunction in AD^40^. Another study suggested that human iPSC-derived human ApoE4-expressing neurons show higher levels of tau phosphorylation than those expressing human ApoE3 in mouse models^41^. Thus, these results are in line with ours: ApoE4 worsens tau pathology. In contrast, Zhao et al. suggested that ApoE2 is associated with increased tau pathology relative to ApoE3 or ApoE4 in primary tauopathies such as progressive supranuclear palsy and corticobasal degeneration^42^. Therefore, the relevance of ApoE isoforms in tauopathy requires further investigation.

ApoE4 is associated with mitochondrial dysfunction, and impaired mitophagy may contribute to mitochondrial dysfunction and lead to AD pathogenesis^39,43,44^. However, the molecular mechanism by which ApoE4 impairs mitochondrial function is not well understood. Here, we observed decreased expression of BNIP3 and PINK1, the key molecules in mitophagy, in ApoE4 carriers compared to ApoE4 non-carriers (Fig. 2D,E). In addition, the phosphorylation level of ubiquitin at Ser65, which is known to be directly phosphorylated by PINK1^17^, was significantly reduced (Fig. 3B,E), suggesting that the activity of PINK1 is highly suppressed in ApoE4 carriers. Thus, our results explain for the first time how ApoE4 evokes mitochondrial and mitophagic dysfunction in AD brains with the *APOE4* allele.

Mitochondrial dysfunction has been strongly implicated in glucose hypometabolism, represented by compromised energy metabolism in the early stages of AD pathology^15,45^. Glucose hypometabolism in the brain is associated with impairment of insulin signaling as well as cognitive decline^15,45^. FDG-PET neuroimaging studies have also shown that cerebral glucose metabolism is reduced in regions associated with AD, especially in ApoE4 homozygote individuals^46,47^. A study using human ApoE4-targeted replacement mice revealed that ApoE4 interferes with neuronal insulin signaling by trapping insulin receptors in the endosomes^48^, explaining how ApoE4 affects the reduction of glucose metabolism in the brain. FoxOs have been shown to play an important role in upregulating genes such as glucose 6 phosphatase (G6Pase) and phosphoenolpyruvate (PEPCK), which control glucose metabolism^49^. Considering the result that ApoE4 represses FoxO3a (Fig. 1), ApoE4 could reduce glucose metabolism via FoxO3a in the brain. In addition, mitochondrial dysfunction could be elicited by ApoE4-mediated downregulation of mitophagy genes such as *BNIP3* and *PINK1*via FoxO3a repression (Fig. 2D,E). Thus, it is inferred that ApoE4 could result in glucose hypometabolism in the brain via various metabolic processes, such as FoxO3a repression and insulin signaling interference.

FoxO3a maintains neural stem cell homeostasis as well as mature neurons. FoxO3a knockout neural stem cells (NSCs) show decreased self-renewal, increased apoptosis, increased oxidative metabolism, and depletion of the NSC pool^50,51^. In addition, FoxO3a directly binds genes involved in the autophagy network through genomic approaches and modulates the induction of autophagy in neural stem and progenitor cells^26^. These findings suggest that FoxO3a is likely to be critical for maintaining neuronal homeostasis during neuronal development. Conversely, defective FoxO3a may induce an imbalance in the neuronal network, eventually leading to neurodegenerative diseases such as AD. According to recent studies, *KLOTHO-VS* heterozygosity is associated with reduced AD risk and β-amyloid burden in individuals carrying the *APOE4* allele^52,53^. However, it remains unclear how it biologically protects ApoE4 carriers from AD. A previous study showed that Klotho reduces oxidative stress by negatively regulating the PI3K/AKT signaling pathway and subsequently enhancing FoxO3a-mediated MnSOD expression in the kidneys of mouse models^54^. In addition, several meta-analyses have shown that *KLOTHO-VS* is significantly associated with exceptional longevity (aged 85 +) along with *APOE* and *FOXO3A*^55,56^. Thus, FoxO3a may be a key player in the health and age-related diseases of the brain.

Our study has some limitations. First, there were no *APOE4* homozygote tissues in ApoE4 carriers. If the experiments, including *APOE4* homozygous samples, had been conducted, the differences in biological processes according to ApoE isoforms could have been more clearly observed. In addition, as the number of ApoE4 carriers was insufficient, comparisons could not be made with the same number of non-carriers. Second, we do not know the biological mechanism by which ApoE4 reduces FoxO3a expression although we have demonstrated an increased interaction between these proteins. Therefore, further studies are needed to elucidate the mechanism by which ApoE4 regulates FoxO3a.

In conclusion, we suggest that ApoE4 may contribute to AD pathogenesis by attenuating autophagy and mitophagy through the repression of FoxO3a. The *APOE* and *FOXO3A* genes have been consistently reported by genome-wide association studies as the two most robust genetic loci associated with longevity across populations^57,58^. However, there was no link between ApoE and FoxO3a in biological processes. Herein, we identified a relationship between ApoE4 and FoxO3a on autophagy in the brain for the first time. Our findings may serve as a novel therapeutic strategy for ApoE4 carriers in AD.

## Methods

### Ethic statement

All participants were recruited from the Seoul National University Hospital Brain Bank (SNUHBB). In all cases, written informed consent for the research was obtained from the patient before death or legal guardian. This study was approved by the Institutional Review Board of the Korea Disease Control and Prevention Agency for using human brain tissue samples (IRB number: 2019-05-02). All experiments were performed following relevant guidelines and regulations.

### Human brain tissue

Human brain tissues were provided from the SNUHBB, where neuropathological diagnosis of the postmortem human brain was performed. The diagnosis of AD followed the diagnostic criteria of the National Institute of Aging and Alzheimer’s Association (NIA-AA) guidelines^59^. The brain tissues of AD patients used in this study were all positive for amyloid and phospho-tau deposition (Supplementary Fig. S1 and Table). Five ApoE4 carriers and seven non-carriers were enrolled in this study. The patient’s epidemiological information was summarized in the Supplementary Table.

### Antibodies

Anti-FoxO3a (2497), phospho-FoxO3a (Ser253) (13129), Atg12 (4280), Beclin-1 (3495), BNIP3 (44060), PINK1 (6946), LC3 (12741), p62/SQSTM1 (8025), phospho-ubiquitin (Ser65) (62802), and optineurin (58981) antibodies were purchased from Cell Signaling Technology. Anti-ApoE4 (MABN43) and β-actin (A5316) antibodies were obtained from Sigma-Aldrich. Anti-tau (A0024) and phospho-tau (Ser262) antibodies were purchased from DAKO and Abcam, respectively. Anti-PHF1 antibody for detecting phosphorylated tau at Ser396/404 residues was described in a previous study^60^.

### Immunoblotting

Human brain tissues (superior frontal gyrus, 200 mg) were homogenized and lysed in 500 μl of RIPA buffer (Cell Signaling Technology) containing 1 mM NaF and 1 × protease inhibitor cocktail (Sigma-Aldrich) on ice. Tissue lysates were harvested by centrifugation at 13,000 rpm for 30 min at 4 °C, and the protein concentration was determined using the Bradford assay (Bio-Rad). Total proteins were separated by NuPAGE 4–12% Bis–Tris Gels (Thermo Fisher Scientific) in MES SDS running buffer (Thermo Fisher Scientific) and then transferred to PVDF membranes (Millipore). The membranes were blocked with 5% skim milk in Tris-buffered saline with 0.1% tween-20 for 1 h and probed with specific antibodies at 4 °C overnight. Immune complexes were detected using horseradish peroxidase-conjugated anti-rabbit or anti-mouse antibodies, followed by enhanced chemiluminescence (ECL, Amersham). For the re-blotting, the membrane was stripped using the Restore Western Blot Stripping Buffer (Thermo Fisher Scientific, 21059) and then incubated with an antibody of interest. The densitometric analyses of the western blots were performed using NIH ImageJ software.

### Statistical analysis

All statistical analyses were performed using GraphPad Prism software (version 9.1.0). *P-*values were calculated using a two-tailed Student’s t-test. Pearson’s correlation analysis was used to measure the degree of association between the two variables. A *p*-value of less than 0.05 was considered statistically significant.

## Supplementary Information

Supplementary Information.
