# Supplementary material for: ApoE4 attenuates autophagy via FoxO3a repression in the brain
Source: Sci Rep. 2021 Sep 2;11:17604. doi: 10.1038/s41598-021-97117-6 (PMC8413297; doi:10.1038/s41598-021-97117-6)
Supplement: Supplementary file 1 — Supplementary Information. [file 41598_2021_97117_MOESM1_ESM.pdf]

ApoE4 attenuates autophagy via FoxO3a repression in the brain

Hee-Young Sohn[1, 3], Seong-Ik Kim[2], Jee-Yun Park[1], Sung-Hye Park[2], Young Ho Koh[1], Joon Kim[3*] and Chulman Jo[1*]

[1]Division of Brain Disease Research, Department for Chronic Disease Convergence Research, Korea National Institute of Health, 187 Osongsaengmyeong2-ro, Osong-eup, Cheongju-si, Chungcheongbuk-do 28159, Republic of Korea

[2]Department of Pathology, Seoul National University College of Medicine, Seoul 03080, Republic of Korea

[3]Laboratory of Biochemistry, Division of Life Sciences, Korea University, Seoul 02841, Republic of Korea

* These authors contributed equally to this work.

E-mail: joonkim@korea.ac.kr; chulmanjo@gmail.com

Supporting Information

Supplementary Materials and Methods

Plasmids

Human ApoE3 cDNA clone (NM_00041) in pCMV6 with C-terminal Myc Tag was purchased from OriGene Technologies. Site-directed mutagenesis was used to generate human ApoE4 (T388C) cDNA clone and confirmed by DNA sequencing. Human FoxO3a plasmid was a gift from Dr. Min-Ju Kim (Hallym University, Korea).

Immunohistochemical staining

Human brain tissue sections were cut into 3 μm thickness for immunohistochemistry, which was carried out on BenchMark ULTRA (Ventana-Roche). For amyloid-β immunostaining, heat-induced antigen retrieval in cell conditioning 1 (CC1) buffer (Roche) was performed at 100°C for 32 min. The tissues were then incubated at 37°C for 32 min with a mouse monoclonal amyloid-β antibody 6E10 (BioLegend, 803001), followed by incubation with a biotinylated secondary antibody at 37°C for 8 min. The staining results were evaluated under a light microscope (Olympus BX40).

Cell culture

HEK293 cells were purchased from the ATCC. Cells were cultured in Dulbecco's modified Eagle's medium (Gibco) supplemented with 10% FBS (Gibco) and 1% penicillin/streptomycin (Gibco) at 37 °C in a humidified atmosphere containing 5% CO_2.

Co-immunoprecipitation

HEK293 cells were transiently transfected with Myc-ApoE3 or Myc-ApoE4 and FoxO3a expression plasmids, for 24 h. Cells were lysed NP-40 lysis buffer (0.5% NP-40, 10 mM Tris-HCl [pH 8.0], 150 mM NaCl, 10 mM sodium pyrophosphate, 1 mM EDTA) containing 1 mM NaF, 1 mM Na_3VO_4, and 1x protease inhibitor cocktail (Sigma-Aldrich). The cell lysates were used for ApoE immunoprecipitation using an anti-Myc antibody (Cell signaling, 2276). The protein level of FoxO3a co-immunoprecipitated with ApoE was examined by immunoblotting using an anti-FoxO3a antibody (Cell signaling, 2497).

Supplementary Table

Supplementary Table The patient's epidemiological information of human brain specimens

No.	Specimens	Age	Sex	PMI(h)	Diagnosis	Braak stage	APOE genotype
1	Non-carrier 1	56	M	3	PART		E3/E3
2	Non-carrier 2	50	M	8	ARTAG		E3/E3
3	Non-carrier 3	75	F	5	AD	III	E3/E3
4	Non-carrier 4	55	M	9	Normal		E3/E3
5	Non-carrier 5	93	F	13	AD	IV	E3/E3
6	Non-carrier 6	61	M	12.5	PART		E3/E3
7	Non-carrier 7	74	M	5.3	PART		E2/E3
8	E4 Carrier 1	75	F	8	AD	V or VI	E3/E4
9	E4 Carrier 2	94	F	7	AD	IV or V	E2/E4
10	E4 Carrier 3	69	M	7	AD	II	E3/E4
11	E4 Carrier 4	83	F	9.5	VD		E3/E4
12	E4 Carrier 5	85	F	7	AD	IV	E3/E4

NOTE. PMI, Post-Mortem Interval; PART, Primary age-related tauopathy; AD, Alzheimer's Disease; ARTAG, Aging-related tau astrogliopathy; VD, Vascular Dementia

Supplementary Figure S1

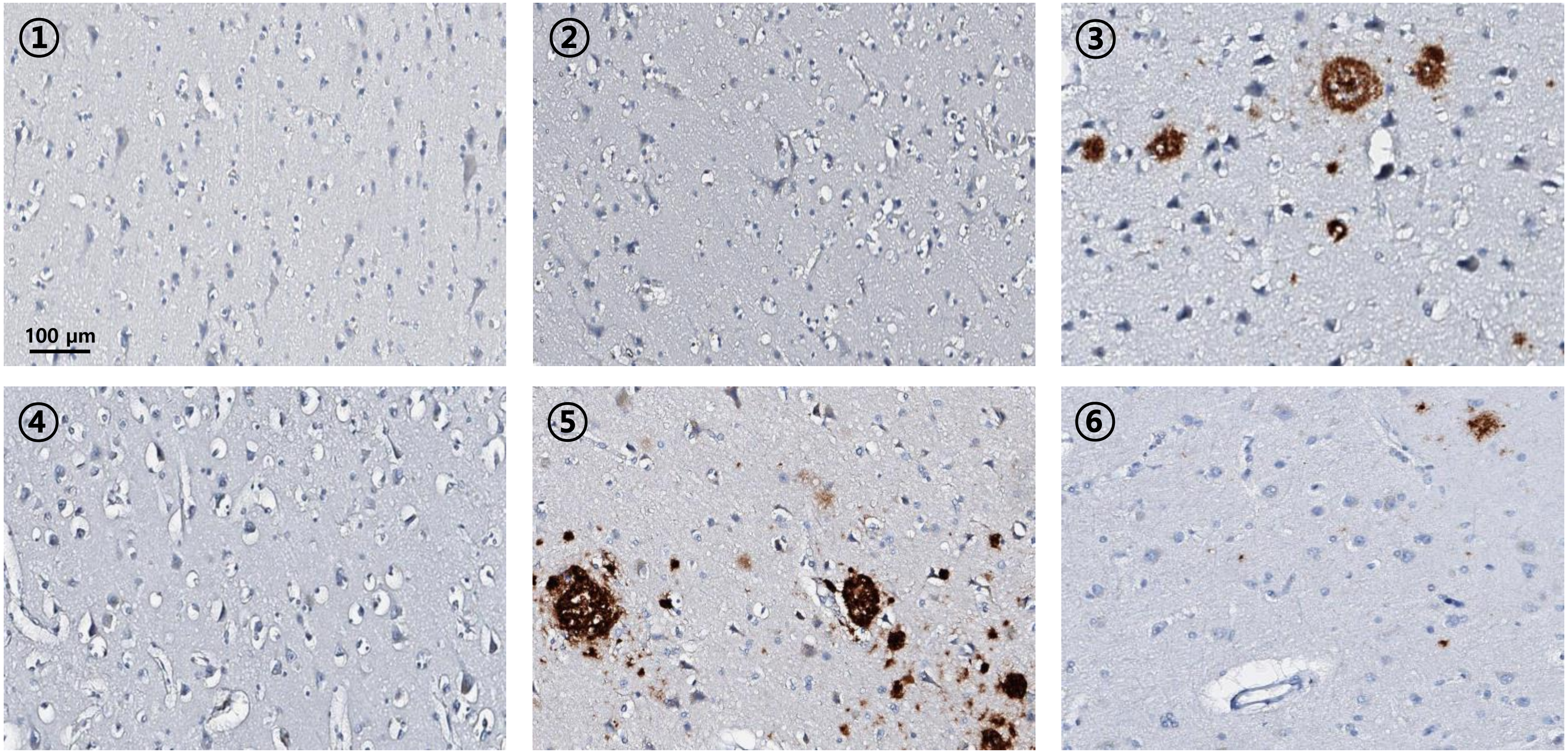

Supplementary Fig. S1. Immunohistochemical staining of β-amyloid plaques in postmortem human brains. Immunohistochemistry analyses were performed on the same brain region (superior frontal gyrus) of ApoE4 non-carriers (① ~ ⑦) and carriers (⑧ ~ ⑫). Scale bars 100 µm.

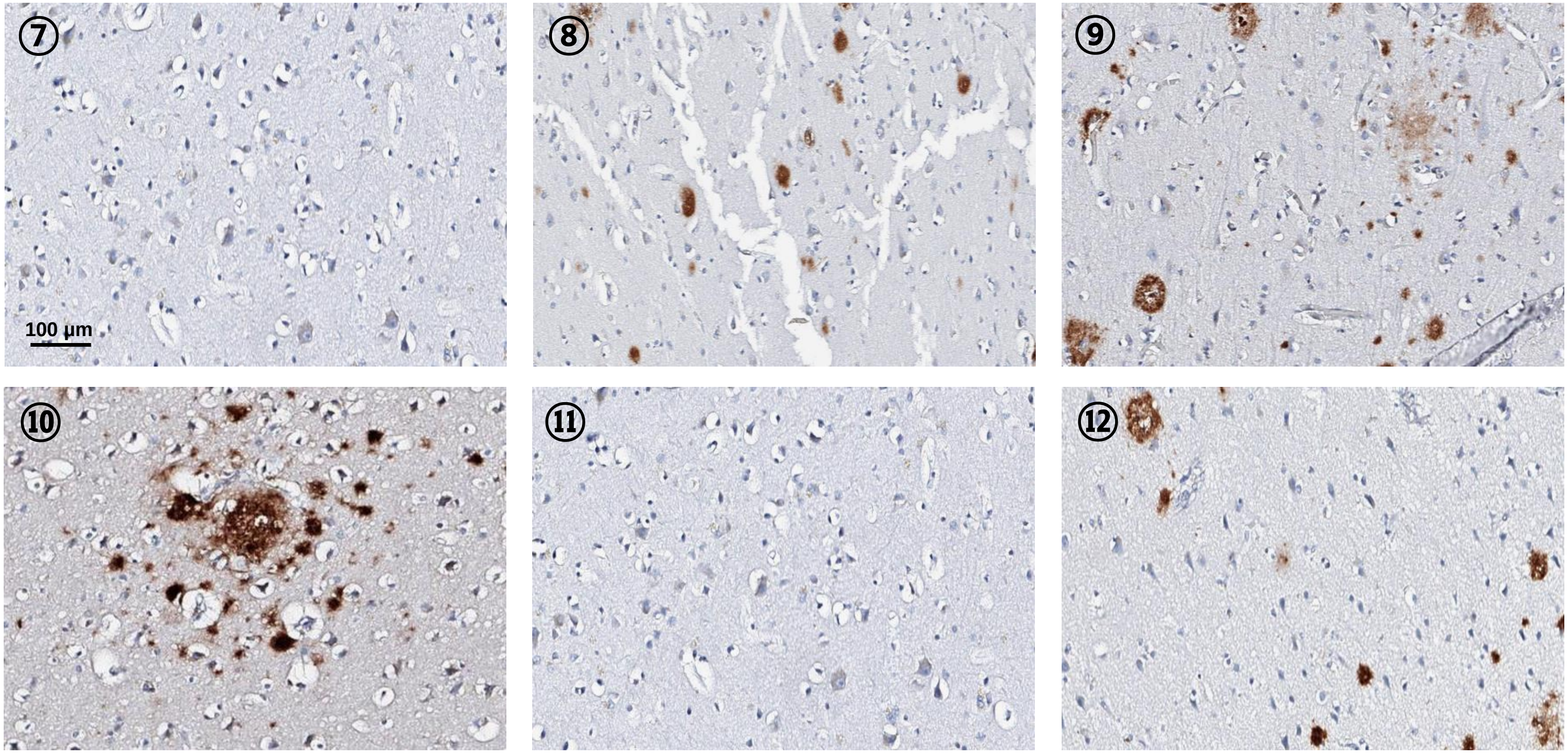

Supplementary Fig. S1 continued

Supplementary Figure S2

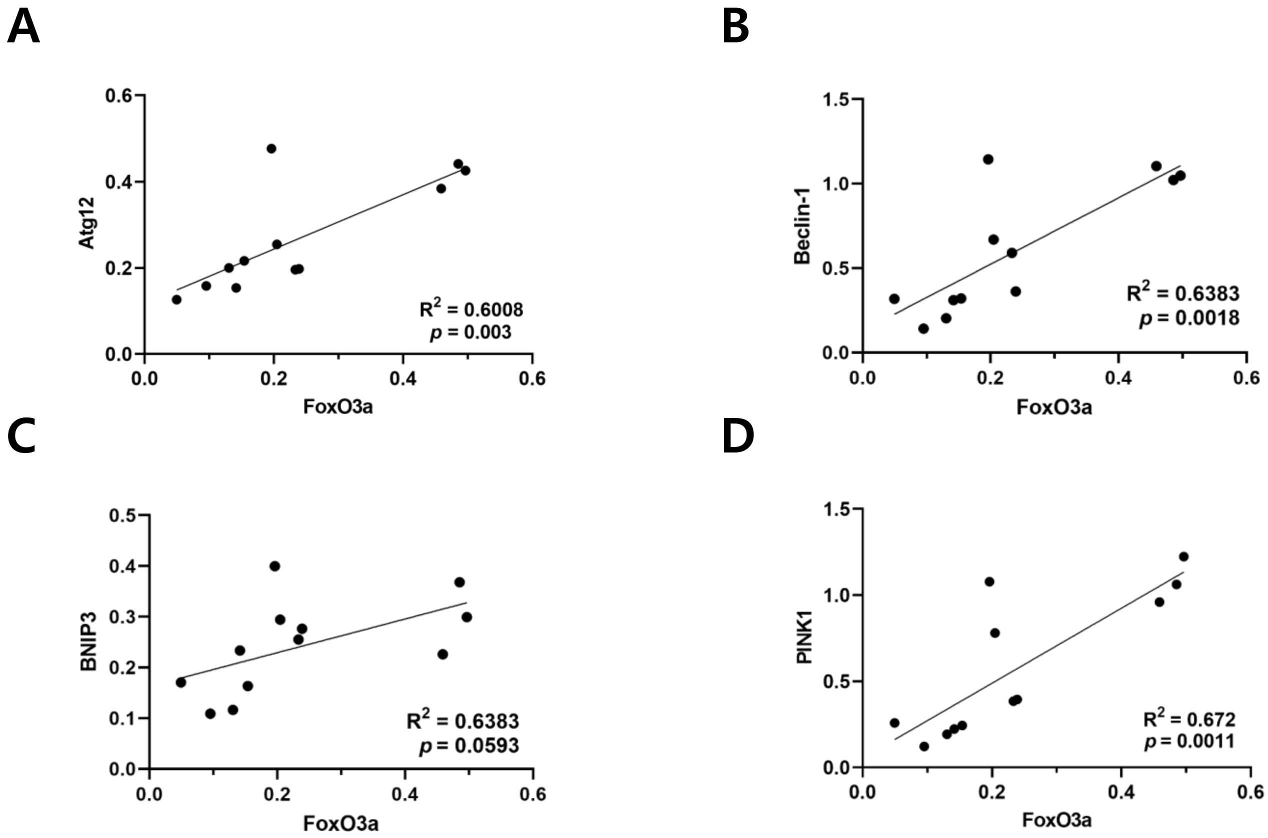

Supplementary Fig. S2. The protein level of FoxO3a was positively correlated with those of its downstream genes. (A) FoxO3a showed positive correlations with Atg12 (R^2 = 0.6008; p = 0.003), (B) Beclin-1 (R^2 = 0.6383; p = 0.0018), (C) BNIP3 (R^2=0.6383; p = 0.0593), and (D) PINK1 (R^2 = 0.672; p = 0.0011). Data were analyzed with Pearson's correlation test (ApoE4 carriers n = 5, non-carriers n = 7). Linear regressions are shown as solid lines.

Supplementary Figure S3

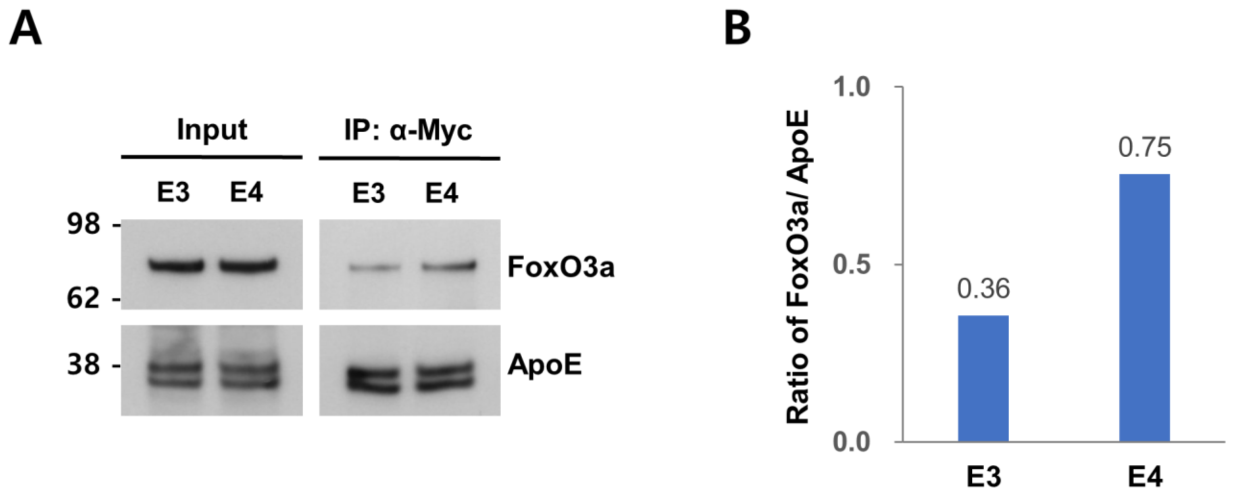

Supplementary Fig. S3. FoxO3a associated with ApoE4. (A) HEK293 cells were co-transfected with Myc-tagged ApoE3 (E3) or ApoE4 (E4) and FoxO3a plasmids. On the next day, the protein levels of FoxO3a co-immunoprecipitated with ApoE were analyzed by immunoblotting. Full blots are provided in Supplementary Fig. S4. (B) Bar graph shows relative ratio of FoxO3a co-immunoprecipitated with ApoE3 or ApoE4.

Supplementary Figure S4

Full-length blots of Fig. 1A

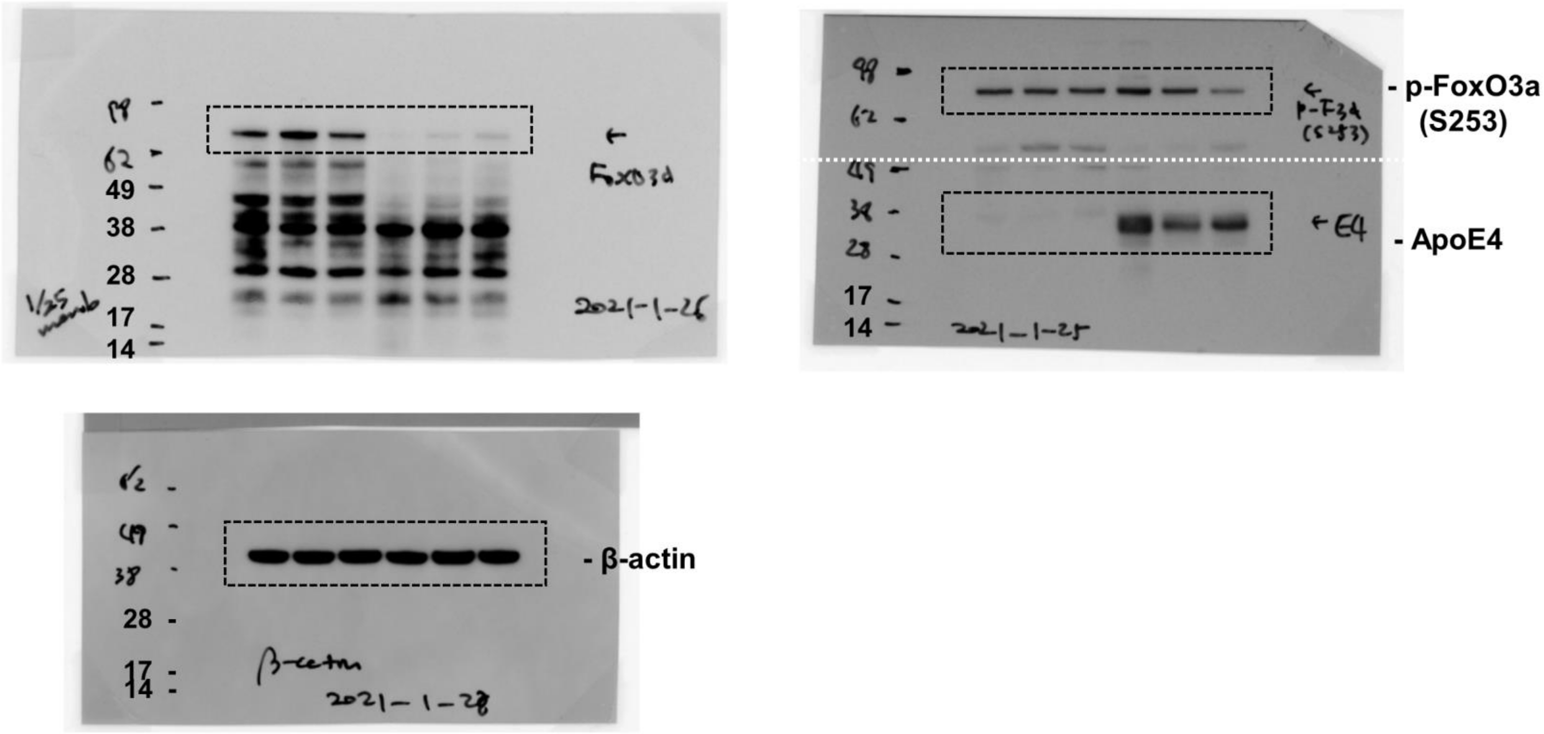

Supplementary Fig. S4. Uncropped immunoblotting images. The dashed boxes indicate the cropped position of membranes in figures. Cutting of membranes for hybridization with different antibodies is indicated by white dotted lines.

Full-length blots of Fig. 2A

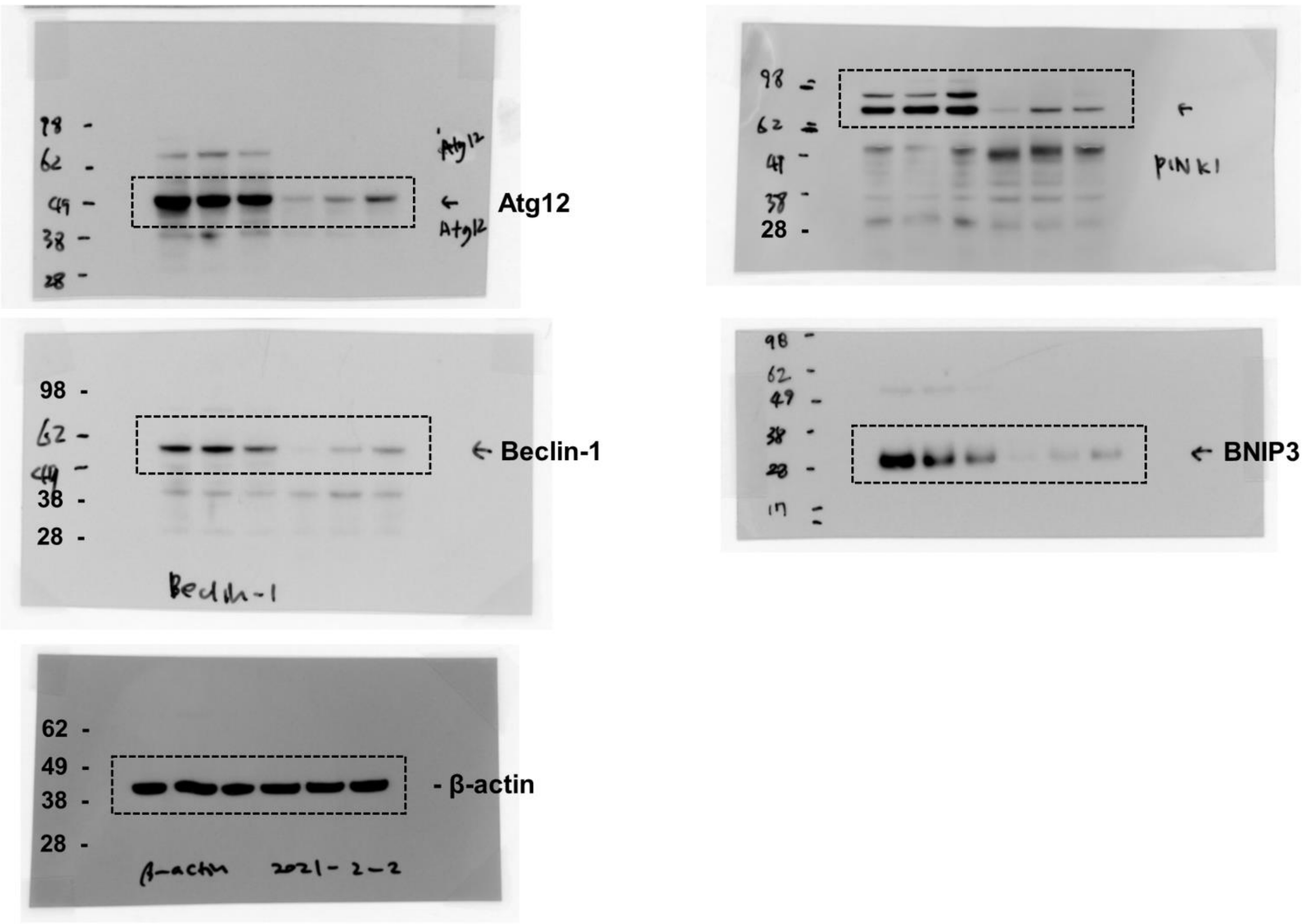

Supplementary Fig. S4 continued

Full-length blots of Fig. 3A

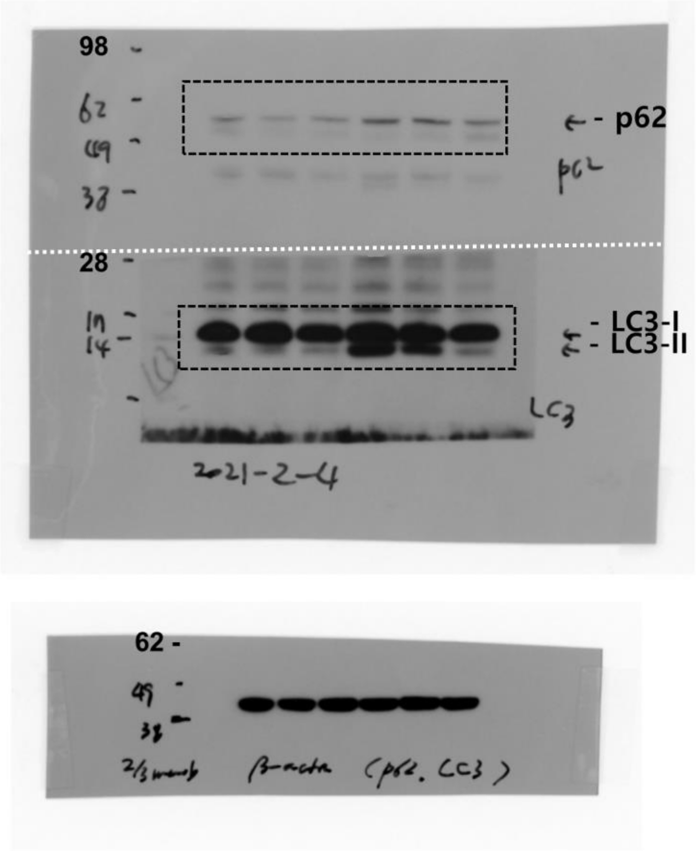

Full-length blots of Fig. 3B

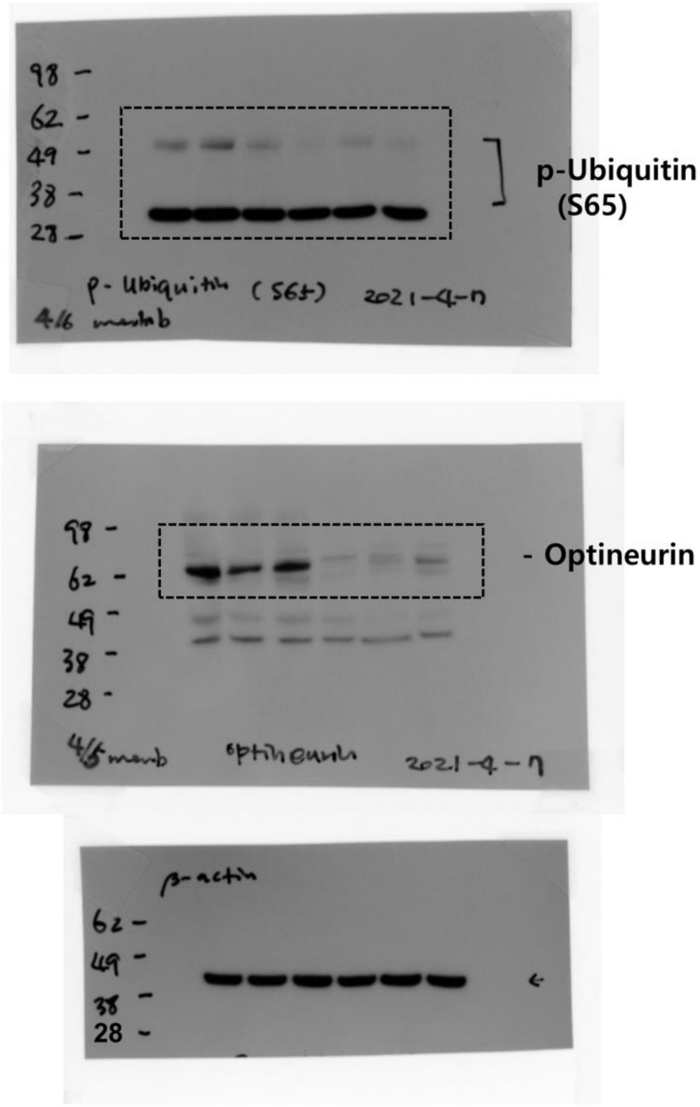

Supplementary Fig. S4 continued

Full-length blots of Fig. 4A

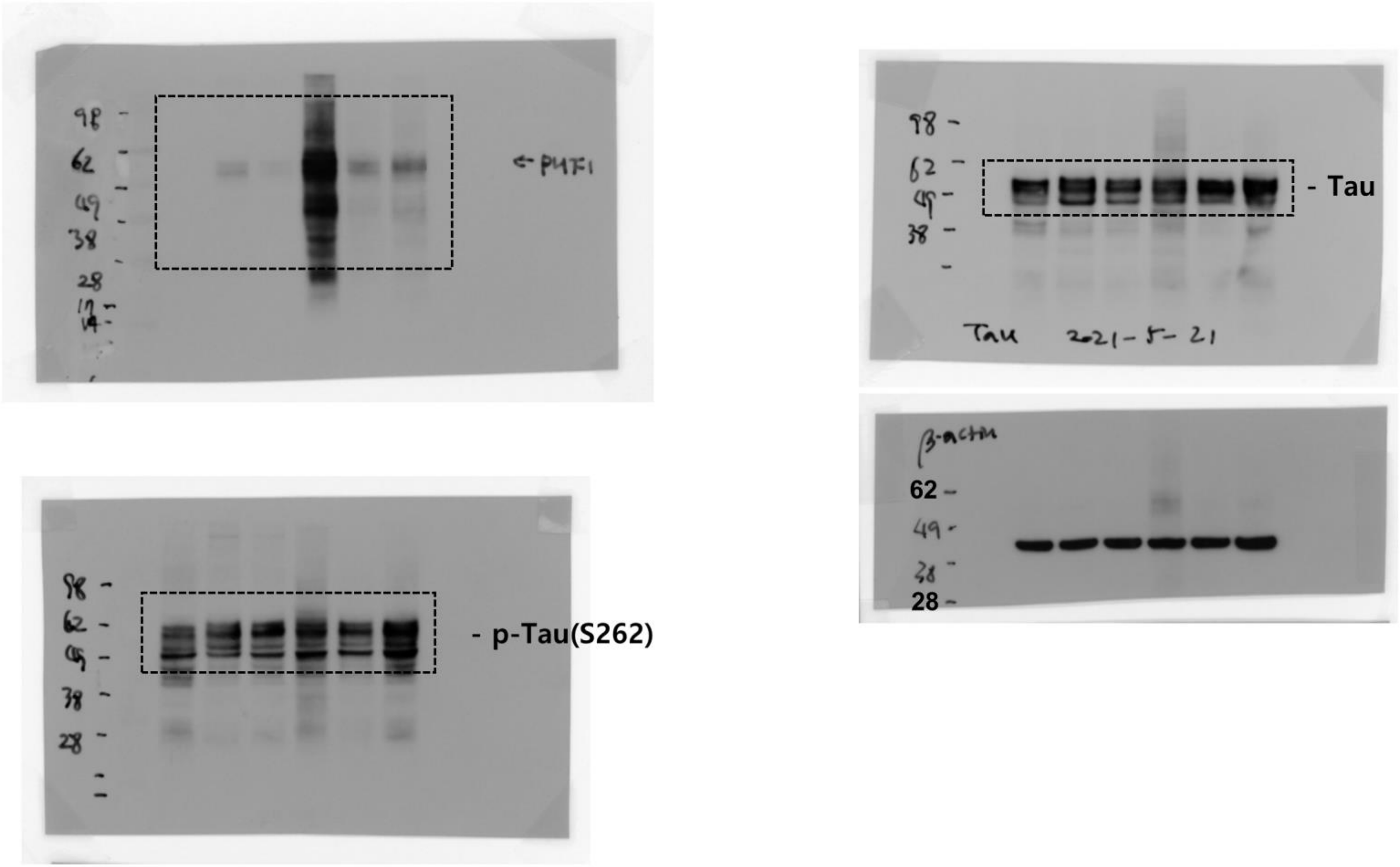

Supplementary Fig. S4 continued

Full-length blots of Fig. S3A

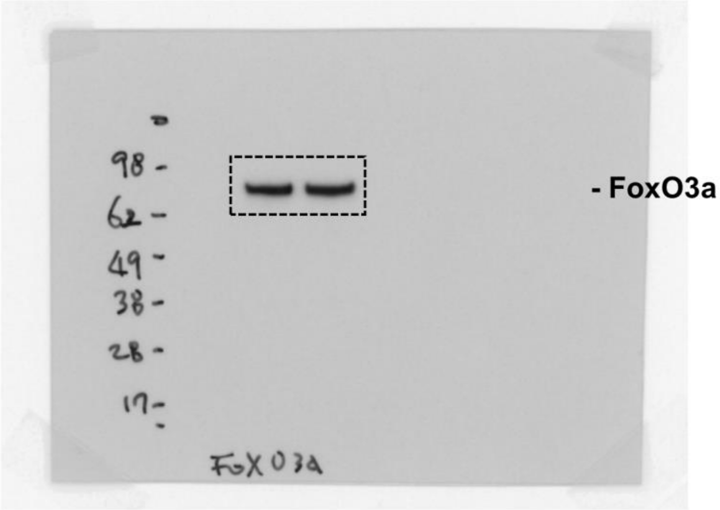

short exposure

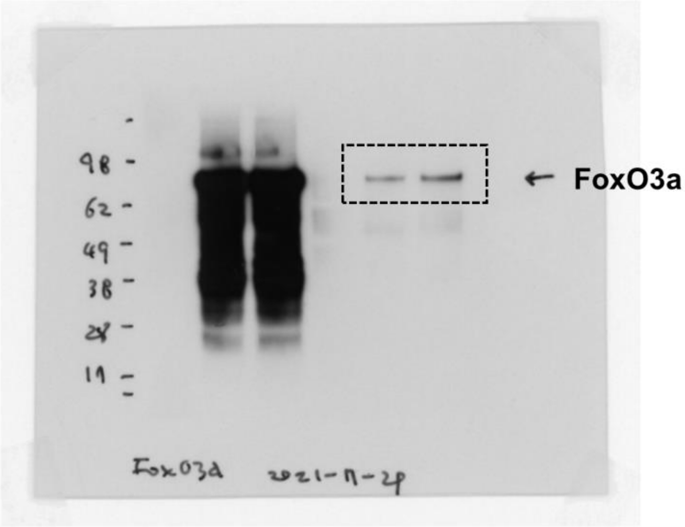

Long exposure

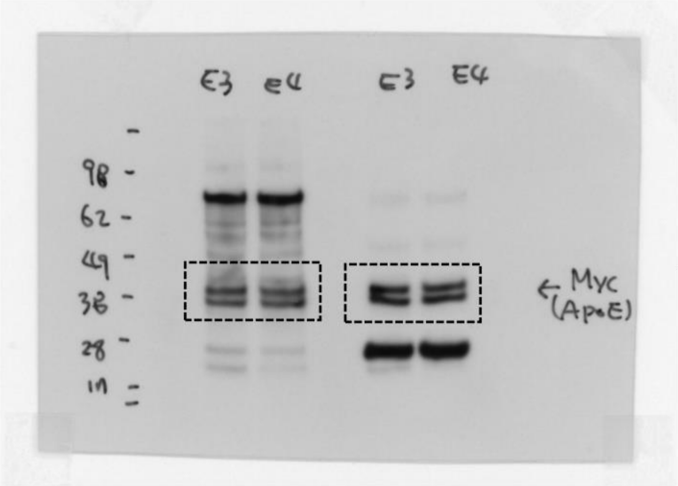

← This blot was reprobed with anti-Myc antibody following FoxO3a immunoblotting using anti-FoxO3 antibody.

Supplementary Fig. S4 continued
